# The Role of Hydrogen in ReRAM

**DOI:** 10.1002/adma.202408437

**Published:** 2024-10-14

**Authors:** Horatio R. J. Cox, Matthew K. Sharpe, Callum McAleese, Mikko Laitinen, Jeevan Dulai, Richard Smith, Jonathan England, Wing H. Ng, Mark Buckwell, Longfei Zhao, Sarah Fearn, Adnan Mehonic, Anthony J. Kenyon

**Affiliations:** ^1^ Department of Electronic and Electrical Engineering University College London Torrington Place London WC1E 7JE UK; ^2^ Ion Beam Centre Advanced Technology Institute University of Surrey, UK Guildford GU2 7XH UK; ^3^ Department of Physics University of Jyväskylä P.O. Box 35 Jyväskylä FI‐40014 Finland; ^4^ Department of Chemical Engineering University College London Torrington Place London WC1E 7JE UK; ^5^ Department of Materials Imperial College London South Kensington Campus London SW7 2AZ UK

**Keywords:** defects, hydrogen, memristor, ReRAM, ToF‐ERDA

## Abstract

Previous research on transistor gate oxides reveals a clear link between hydrogen content and oxide breakdown. This has implications for redox‐based resistive random access memory (ReRAM) devices, which exploit soft, reversible, dielectric breakdown, as hydrogen is often not considered in modeling or measured experimentally. Here quantitative measurements, corroborated across multiple techniques are reported, that reveal ReRAM devices, whether manufactured in a university setting or research foundry, contain concentrations of hydrogen at levels likely to impact resistance switching behavior. To the knowledge this is the first empirical measurement depth profiling hydrogen concentration through a ReRAM device. Applying a recently‐developed Secondary Ion Mass Spectrometry analysis technique enables to measure hydrogen diffusion across the interfaces of SiO_x_ ReRAM devices as a result of operation. These techniques can be applied to a broad range of devices to further understand ReRAM operation. Careful control of temperatures, precursors, and exposure to ambient during fabrication should limit hydrogen concentration. Additionally, using thin oxynitride or TiO_2_ capping layers should prevent diffusion of hydrogen and other contaminants into devices during operation. Applying these principles to ReRAM devices will enable considerable, informed, improvements in performance.

## Graphical abstract

Quantitative measurements reveal high concentrations of hydrogen across many Redox‐based resistive random access memory (ReRAM) devices, exceeding critical thresholds. Secondary Ion Mass Spectrometry confirms that this hydrogen diffuses across the reactive interface under electrical biasing, indicating a significant impact on resistive switching. These findings highlight the necessity of incorporating hydrogen dynamics into ReRAM models and the importance of precise impurity control during fabrication to optimize device performance.

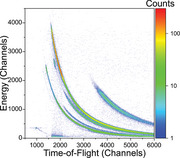

## 1 Introduction

Redox‐based resistive random access memory (ReRAM) has recently attracted significant attention as a next‐generation non‐volatile memory and a crucial memristive component in emerging neuromorphic computing systems. Such systems hold promise for advancing AI‐driven tasks such as image recognition, semantic analysis, voice recognition, and autonomous driving, as well as enabling complex computations in self‐driving vehicles, robotics, biomedical data processing, and wearable technologies.^[^ ^1^ ^]^ ReRAM devices typically comprise a highly insulating thin film oxide layer sandwiched between two electrodes. Our focus is on filamentary valence change (intrinsic) memories in which applying a small voltage across the nanometres‐thick oxide drives oxygen diffusion, the formation and disruption of oxygen vacancy filaments, and hence modulates device resistance.^[^ ^2^ ^]^

Reliability is a pressing issue for ReRAM. There can be significant performance variability between identically fabricated devices, the cause of which is not yet fully understood.^[^ ^3^ ^]^ If ReRAM devices are to be integrated into larger‐scale computing systems it is essential to understand the root cause of this variability. It is important to remember that similar issues of variability and reliability were faced in the development of transistors^[^ ^4^ ^]^ and it is only through intensive research that these problems were progressively solved.^[^ ^5^ ^]^ Crucially, this research followed a materials‐based approach, with extensive empirical measurements of device microstructure and composition. The same approach is needed for ReRAM technologies. Thus far, switching material choice and microstructure have been studied extensively, leading to great progress in performance.^[^ ^6^, ^7^, ^8^, ^9^, ^10^ ^]^ It is time to extend this research to empirical measurements of the effect of impurities, in particular hydrogen. While the effect of hydrogen on performance remains relatively unexplored, it should not be discounted it is a highly mobile and reactive species present in ReRAM devices that rely on reactive, mobile ions for operation. Thus, the open questions are: how much hydrogen is present in devices, can it be controlled, and what role does it play in resistance switching?

### 1.1 The Role of Hydrogen in Gate Dielectrics

Many of the answers can be found in the field of gate dielectrics, as resistance‐switching oxides and transistor gate oxides are similar. A large body of research has shown that hydrogen plays a key role in the time‐dependent dielectric breakdown of gate oxides. Hydrogen can generate defects crucially, oxygen vacancies.^[^ ^11^, ^12^ ^]^ The migration of hydrogen under an applied field is strongly correlated with oxide degradation.^[^ ^13^, ^14^ ^]^ Consequently, under applied voltage stress, trap‐assisted tunneling through defects generated by hydrogen enables stress‐induced leakage current (SILC) to flow, which accelerates gate oxide degradation.^[^ ^15^ ^]^ Over time, increased defect concentration leads to the creation of conductive percolation paths and oxide breakdown.^[^ ^16^ ^]^ If this sounds familiar it is because this is almost identical to the proposed mechanism of soft dielectric breakdown proposed in filamentary ReRAM devices.^[^ ^2^, ^17^ ^]^ Given the findings in the gate oxide community outlined above, hydrogen should therefore also be expected to play a role in ReRAM operation.

### 1.2 The Role of Hydrogen in ReRAM

While transistors employ pure and stoichiometric gate oxides to minimize current leakage and hard breakdown, ReRAM exploits sub‐stoichiometric and leaky oxides to enable reversible resistance switching via soft breakdown. While hydrogen can be detrimental to gate dielectrics creating defects and lowering the breakdown barrier it could be beneficial in ReRAM devices: lowering the breakdown barrier enables low switching voltages. For example, hydrogen plasma exposure of ZnO and HfO_2_ thin films enables improved ReRAM devices with multilevel resistance states, electroforming‐free operation, and a larger resistance window between states.^[^ ^18^, ^19^, ^20^ ^]^ However, in all cases, changes in the electronic properties of the oxide have been attributed only to the hydrogen plasma acting as reducing agent generating increased oxygen vacancy concentration in the near‐surface oxide. Though Photoluminescence Spectroscopy, Auger Electron Spectroscopy, and X‐ray Photoelectron Spectroscopy (XPS) are all used to confirm the changes in oxygen stoichiometry post‐hydrogen plasma treatment they may not provide the full picture. Very similar work in the gate oxide community exposing SiO_2_ thin films to a hydrogen plasma suggests an active role for the ingrained hydrogen species which, depending on conditions, can both passivate electronically active defects and diffuse, leading to an increase in the SiO_2_/Si interface state density.^[^ ^11^, ^12^ ^]^ The tendency in the ReRAM field to focus only on oxygen vacancies neglects the important role of hydrogen.

Clear evidence of this role can be seen in the work of Wei et al. who exposed a TaO_x_ ReRAM device to hydrogen gas, measuring a sharp and consistent increase in oxide conductivity with as little as 1 vol.% of hydrogen in air.^[^ ^21^ ^]^ The device in question is so sensitive to the presence of hydrogen it is being proposed for use as a hydrogen sensor. Significantly, exposure of a pristine TaO_x_ device to hydrogen lowered the oxide resistance; this effect persisted even after the hydrogen gas was removed. Again, parallels can be drawn with gate dielectrics where exposure to hydrogen has been shown to cause leakage currents like those observed after high field stressing.^[^ ^12^ ^]^ In both cases hydrogen exposure modifies the resistance of the oxide permanently.

Beyond the work described above evidence of the impact of hydrogen on ReRAM is less direct, such as the measured impact of ambient humidity on the programming voltages of different devices.^[^ ^22^, ^23^, ^24^ ^]^ Though this is generally attributed to counter‐reactions with hydroxide ions at the electrodes, proton conduction has been suggested as contributing to operation at higher relative humidity levels.^[^ ^25^ ^]^ However, the effect of the ambient environment is only observed for moisture; nitrogen or oxygen partial pressures have negligible effect on the conductivity of ReRAM devices.^[^ ^26^ ^]^ This discovery is a significant advance in addressing ReRAM reliability and consistency. Ambient humidity levels vary from day to day and from lab to lab. However, empirical measurements of hydrogen are lacking; thus, the potential for hydrogen to increase conductivity by generating oxide defects is often overlooked. An exception is the work of Lubben et al. who estimate the residual ‐OH concentration in SiO_2_ thin films exposed to various humidity levels by measuring the SiO_2_ permittivity.^[^ ^27^ ^]^ Though insightful, the study only measures one form that incorporated hydrogen could take, is not quantitative, and only provides relative estimates of ‐OH concentration, illustrating the challenges faced when measuring hydrogen content.

Overall, despite evidence from both the gate oxide and ReRAM communities that oxide electrical conductivity is sensitive to the presence of hydrogen, to our best knowledge no‐one has quantified hydrogen concentration in ReRAM devices. This matters because hydrogen contamination during fabrication is notoriously difficult to avoid. Hydrogen is present in many precursors and solvents,^[^ ^28^, ^29^ ^]^ to some extent in all vacuum systems,^[^ ^30^ ^]^ and is highly soluble in many of the materials used as sputtering targets.^[^ ^31^ ^]^ Furthermore, after fabrication, protons can penetrate oxides from ambient water vapor.^[^ ^32^ ^]^ In 2004 Norby et al. noted that: “Hydrogen, nominally not a component of oxides, is often assumed absent and neglected when considering oxide materials and their properties … But not only is it present, normally as protons, but it also dominates the properties of many oxides, even at high temperatures”.^[^ ^32^ ^]^ The reason it is often neglected is the significant challenge of measuring hydrogen concentration reliably. Nevertheless, it is essential to quantify hydrogen concentration in ReRAM devices. In this work we set out to do this: measuring the hydrogen concentration in a broad range of silicon oxide devices, fabricated both at a university and at a research foundry.

### 1.3 The Threshold Hydrogen Concentration

As a first step, we need to establish a threshold concentration at which hydrogen impurities will modify oxide conductivity significantly. Turning to the gate oxide field where abundant measurements are available we see that, depending on the concentration and distribution of hydrogen, very different effects on the conductivity of oxides can be seen. For example, on implanting hydrogen at maximum concentrations of ≈8 at.% into a ZnO thin film Kennedy et al observed a decrease in the oxide conductivity of almost an order of magnitude.^[^ ^33^ ^]^ This is thought to be due to the implanted interstitial hydrogen passivating deep donor and acceptor states. However, when the implanted films were annealed at 700 °C, their conductivity increased to be three times greater than that of both annealed and pristine ZnO films without H implantation. This was corroborated by other work in which similar increases in conductivity were observed for H implanted ZnO films annealed at 200 °C.^[^ ^34^ ^]^ In this latter study, above a certain hydrogen doping threshold, annealing was not required to increase the conductivity of the oxide. In both cases the net increase in conduction after implantation and annealing is attributed to the movement of hydrogen from an interstitial site to a neighboring oxygen site where it creates a donor state. Thus, hydrogen doping can have opposite effects on the conductivity of an oxide depending on its concentration and position within the lattice. Crucially, annealing at temperatures as low as 200 °C can change the effect of hydrogen from passivating defects to introducing defect states and increasing conductivity. Across various ReRAM systems, localised heating greatly exceeding these temperatures has been predicted and observed during the generation of the conducting filament (electroforming) and subsequent device operation.^[^ ^35^, ^36^, ^37^ ^]^ It is plausible that some contribution to the filament conductivity, and its subsequent modulation, comes from hydrogen‐associated defects.

Even when not intentionally introduced into oxides, hydrogen can modify their electrical properties. In thin films of as‐deposited CVD SiO_2_ gate oxide, peak concentrations of hydrogen have been measured at ≈2 at.% by Nuclear Reaction Analysis (NRA). Annealing reduced the concentration of hydrogen at the cathodic interface, suppressing the generation of bulk defects and reducing field‐induced degradation of oxide resistance.^[^ ^13^, ^38^ ^]^ In contrast, applying a field across SiO_2_ gate dielectric diffuses hydrogen to the Si/SiO_2_ interface, increasing interface state density and reducing device stability.^[^ ^39^ ^]^ Thus, even at peak concentrations as low as ≈2 at.%, hydrogen diffusion modifies measurably the electronic properties of SiO_2_.

An extensive analysis of the effects of annealing various SiO_2_ films deposited on Si wafers revealed: a high hydrogen concentration of up to ≈8 at.% in as‐deposited oxides, a consistent reduction in hydrogen content with annealing, and a subsequent increase in hydrogen content when annealed films were exposed to ambient.^[^ ^40^ ^]^ Capacitance‐voltage (*C–V*) measurements of samples revealed large positive and negative flatband voltages directly correlated with hydrogen concentration. From this, the authors suggest the existence of a critical hydrogen concentration for optimal oxide electrical properties. While the desired properties will be different for ReRAM, a similar critical hydrogen concentration may exist. Given this, as for gate oxides, hydrogen diffusion from the ambient could explain degradation of ReRAM performance over time.^[^ ^41^ ^]^ The most common ReRAM degradation is the loss of the high resistance state (HRS), which becomes increasingly conductive with use.^[^ ^42^ ^]^ This could be a product of increasing hydrogen concentration in devices over their lifetime, as seen in gate oxides.

Thus, depending on concentration and distribution, hydrogen can passivate or introduce donor states, leading to decreases or increases in oxide conductivity as well as negative or positive flatband voltages. Annealing reduces oxide hydrogen concentration; exposure to ambient increases it; under an electric field hydrogen diffuses, increasing interface state density and degrading the oxide. Measurable changes in electrical properties are consistently observed in SiO_2_ for changes in H concentration of ≤2 at.%, a concentration at which it is reasonable to expect that hydrogen will play a role in resistance switching.

### 1.4 How to Measure the Concentration of Hydrogen

**Figure 1:**
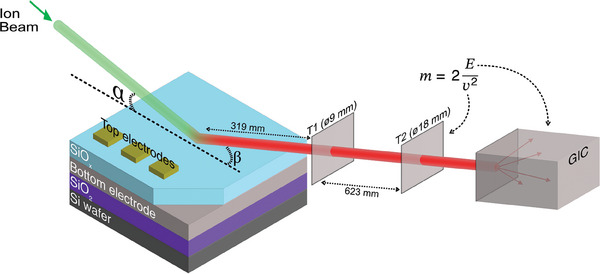
Schematic of the samples and the ToF‐ERDA analysis applied at the University of Jyväskylä, α/β = 20.5°. The ion beam analysis is done in a clear region of SiOx, away from the Au/Ti top electrodes to prevent interference. A high energy (MeV) beam of primary ions (green beam) is used to irradiate the sample surface at a glancing angle, α. This elastically forward recoils sample atoms (red beam) at angle β into two‐timing detectors and a gas ionization chamber, which measure the speed and energy of the atoms, respectively. This enables the determination of the element ejected and its original depth within the material.

Measuring hydrogen concentration presents profound challenges as it is light, mobile, and prevalent in vacuum systems. X‐ray‐based techniques also cannot be used to directly measure its concentration, as hydrogen has no core electrons. To overcome these issues, we used a relatively new technique: Time‐of‐Flight Elastic Recoil Detection Analysis (ToF‐ERDA), as shown in **Figure** **1** for the setup at the University of Jyväskylä. High energy incident ions forward recoil atoms elastically from the sample into time‐of‐flight detectors, T1 and T2, and gas ionization chamber measuring their velocity and energy respectively.^[^ ^43^, ^44^ ^]^ From this the element and depth of origin in the material can be determined with enhanced mass resolution.

ToF‐ERDA is one of very few techniques capable of quantitatively measuring hydrogen concentration alongside the concentration of other elements in the switching layer of our devices. However, it can only profile accurately elements whose atomic weight is lower than that of the impinging ion, which in this work is either chlorine or iodine. Thus, we combine ToF‐ERDA results with those from Rutherford Backscattering Spectrometry (RBS), which measures heavier elements.^[^ ^45^ ^]^ We can then provide a robust measure of the concentration of every element in our ReRAM devices. In combining techniques, our results have a concentration uncertainty of ± 5% of the measured value (e.g., 5 ± 0.25 at.%) for every element apart from hydrogen, for which the uncertainty is ±10% (e.g., 5 ± 0.50 at.%). However, the combined ToF‐ERDA and RBS analysis carried out here requires the assumption of flat interfaces between the thin films during the fitting process. Therefore, complimentary Secondary Ion Mass Spectrometry (SIMS) and XPS measurements were also carried out under different conditions to provide a more complete understanding of the system.

## 2 Results and Discussion

### 2.1 Sample Details

The samples studied are detailed in **Table** **1** below. Further details of their fabrication are given in Section 5: Methods.  

**Table 1:** Samples were analyzed in this study. All samples were grown on an Si wafer with 1um of thermal SiO_2_ and all layers are listed from sample top to bottom. In active ReRAM devices, a Au(100 nm)/Ti(5nm) top electrode is deposited typically on top of the SiO_x_ as shown in Figure 1. Deposition methods were: Reactive magnetron sputtering, Atomic layer deposition (ALD), spin‐on hydrogen silsesquioxane (spin‐coated), Thermal evaporation.).

Sample 1 [ALD]	Sample 2 [UCL41]	Sample 3 [UCL47Pt]	Sample 4 [UCL47Mo]
Au (evaporated): 100 nm Ti (evaporated): 5 nm SiO_x_ (ALD): 30 nm Mo (sputtered): 100 nm	SiO_x_ (sputtered): 26 nm Mo (evaporated): 65 nm	SiO_x_ (sputtered): 12.5 nm Pt (evaporated): 75 nm Ti (sputtered): 5 nm	SiO_x_ (sputtered): 12 nm Mo(evaporated): 75 nm

Sample 5 (UCL47Ti)	Sample 6 (HSQ)	Sample 7 (UCL40Mo)	Sample 8 (UCL40Ti)
SiO_x_(sputtered): 12.5 nm Ti (sputtered): 70 nm	SiO_x_ (spin coated): 45 nm Mo (sputtered): 200 nm	SiO_x_ (sputtered): 12 nm Mo (evaporated): 65 nm	SiO_x_ (sputtered): 10 nm Ti (sputtered): 50 nm

Sample 9 (UCL42)	Sample 10 (UCL49Ti)	Sample 11 UCL54	Sample 12 (INDD4)
Au (evaporated): 100 nm Ti (evaporated): 5 nm SiO_x_ (sputtered): 25 nm Mo (sputtered): 200 nm	SiO_x_ (sputtered): 11 nm Ti (sputtered): 45 nm Au (sputtered): 100 nm Ti (sputtered): 5 nm	Au (evaporated): 100 nm Ti (evaporated): 5 nm SiO_x_ (sputtered): 30 nm Mo (sputtered): 100 nm	TiN (sputtered): 60 nm Ru (sputtered): 2 nm TaN (sputtered): 5 nm

Sample 13 (INDD10)	Sample 14 (INDD11)	Sample 15 (INDD14)	Sample 16 (SiWafer)
TiN (sputtered): 60 nm Ru (sputtered): 2 nm TaN (sputtered): 5 nm	TiN (sputtered): 20 nm Ru (sputtered): 2 nm TaN (sputtered): 5 nm	TiN (sputtered): 20 nm Ru (sputtered): 2 nm TaN (sputtered): 5 nm	SiO_2_ (thermal): 1 µm Si (wafer): 350 µm

### 2.2 Combined ToF‐ERDA and RBS (IBA)

**Figure 2:**
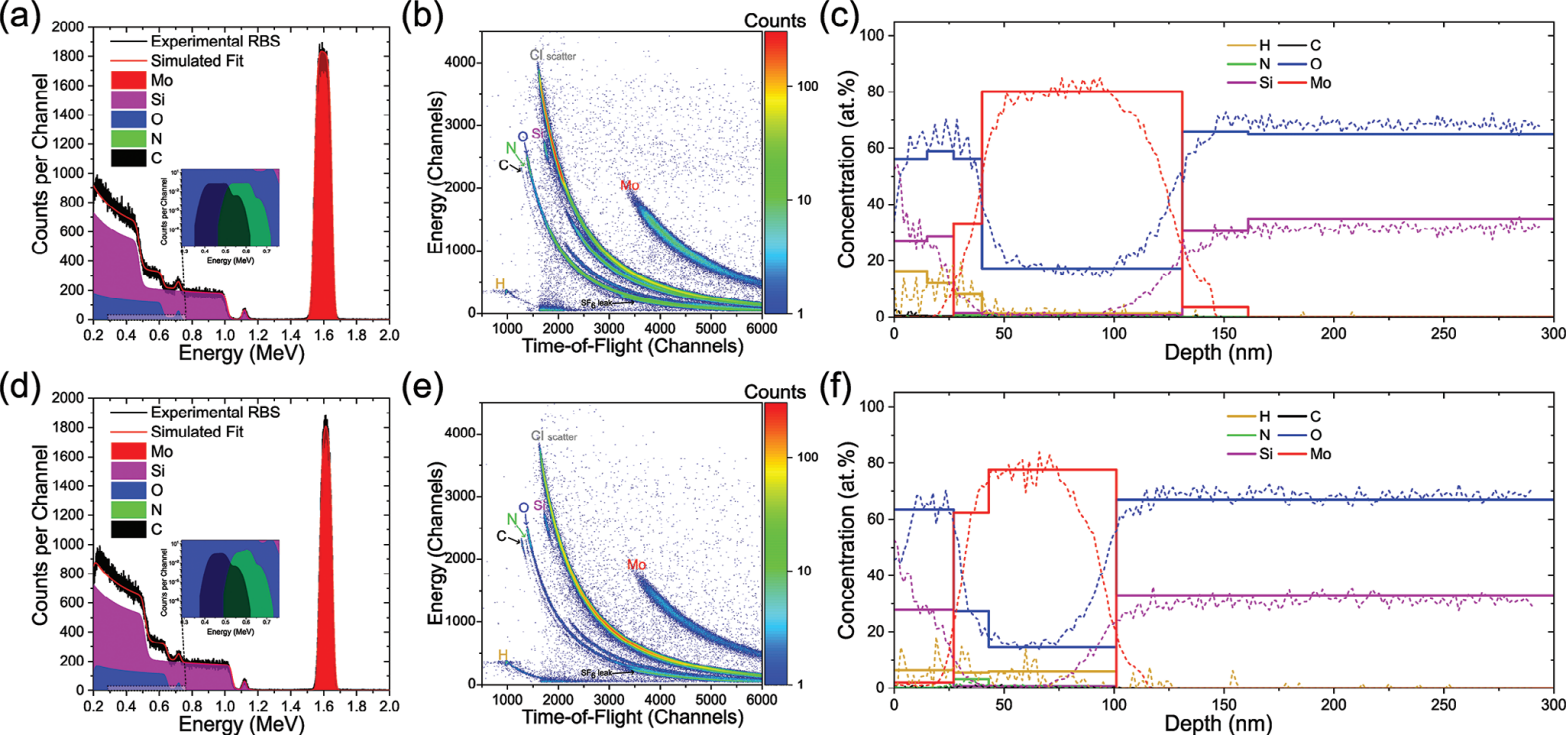
IBA measurements of two samples with nominally the same composition, with a similar thickness of SiO_x_ deposited using a)‐c) Sample 1 – ALD and d)‐e) Sample 9 ‐Sputtering, onto Mo bottom electrodes. a,d) RBS spectra for the sample confirm the concentration of heavier elements. b,e) ToF‐ERDA spectra for both samples, used to generate initial depth profiles (dotted lines) in (c,f). For these depth profiles, concentrations of heavier elements are generated from the beam scatter and are not as accurate due to extended tails from measurement artefacts such as multiple scattering. The solid lines in (c,f) show the final sample composition generated by fitting the ToF‐ERDA results from MCERD to RBS measurements, giving a reliable measure of composition for the full range of elements. For both samples, significant concentrations of H, C, and N impurities were measured, along with oxidation of the Mo electrodes.

The quantitative analysis process is as follows: First, samples are measured with ToF‐ERDA, and the collected histograms shown in **Figure** **2**b,e are analyzed using the software package Potku, which accounts for the loss of lighter elements during measurement and “rolls‐back” their composition to an earlier time in the measurement at the expense of a loss of some statistics.^[^ ^46^ ^]^ From this, the depth profiles shown in Figure 2c,f (dotted lines) are generated. Next, using the energy spectra for each element, MCERD software simulates scattering during analysis and accounts for multiple scattering and other experimental effects. The experimental energy spectra for each element are then fitted, producing a more reliable depth profile.^[^ ^47^ ^]^ Finally, RBS is carried out on the samples shown in Figure 2a,d, and data is analyzed in SIMNRA software.^[^ ^48^ ^]^ Thicknesses and compositions calculated using MCERD from the ToF‐ERDA analysis are input into SIMNRA and used to fit the RBS data using an assumed density for each layer, calculated with SRIM in the case of Mo.^[^ ^49^ ^]^ This final stage provides slight adjustments to layer thicknesses and compositions of heavier elements. The combined results, giving the final composition, are shown Figure 2c,f (solid lines). The combined analysis process detailed above will be described as “Ion Beam Analysis” (IBA) from here on.^[^ ^50^ ^]^

**Figure 3:**
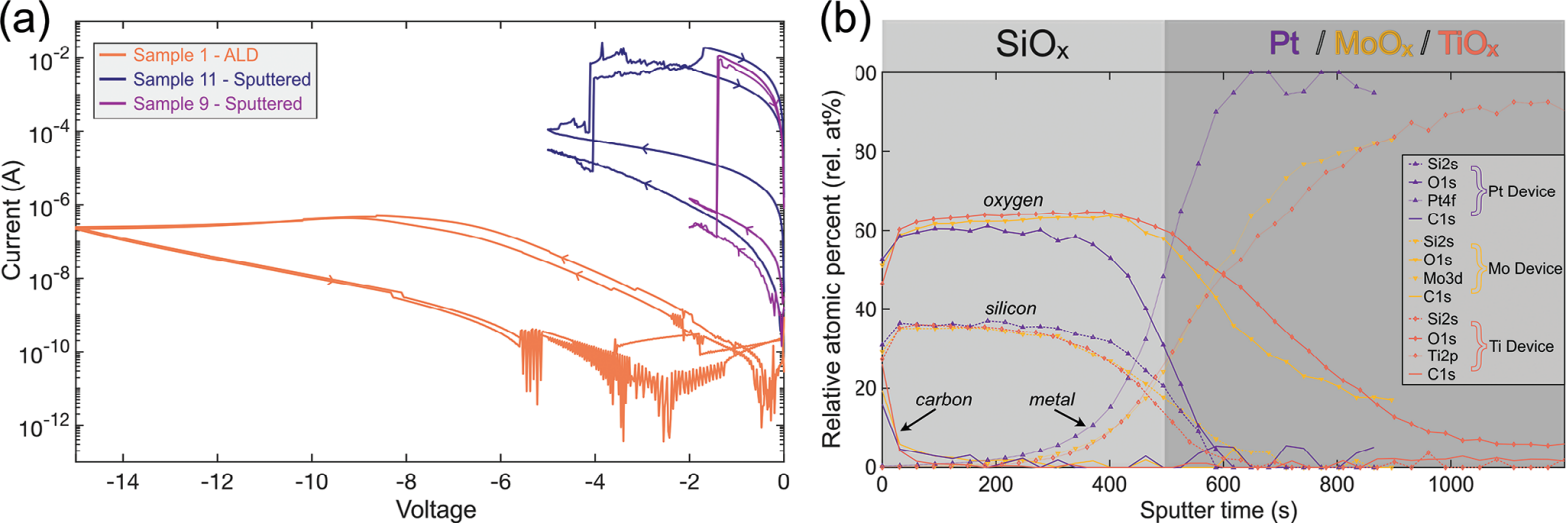
a) Electroforming IV sweeps for two devices of each type. Neither of the two Sample 1 – ALD devices electroform, even at ‐15 V, as the film is relatively defect‐free. Sample 11‐ Sputtered has identical top and bottom electrodes to sample 1 but with a sputtered SiO_x_ layer which electroforms at ≈−5 V in both measured devices. Sample 9 – Sputtered has a thinner sputtered SiO_x_ which is deposited onto a rougher Mo bottom electrode, hence both devices electroformed at much lower voltages of under −2V. b) XPS depth profiles of samples 3, 4, and 5, each with an SiO_x_ layer deposited onto Pt, Mo, and Ti bottom electrodes, respectively. XPS sputtering proceeds completely through the SiO_x_ into the electrode as demonstrated by the drop in measured Si concentration to zero for all three samples at ≈600 s. The location of the SiO_x_/electrode interface is taken to be at the half mark of the drop in Si concentration, illustrated by the contrast between the two sections. The inert Pt electrode has negligible oxidation, while the more reactive Mo and Ti electrodes are significantly oxidized. The survey spectra at three depths through this profile are shown in Figure S14 (Supporting Information). Adapted from^[^ ^41^ ^]^ with permission of the author.

Figure 2 shows the IBA process for sample 1, in which SiO_x_ was deposited onto an Mo bottom electrode using Atomic Layer Deposition (ALD), and sample 9, in which SiO_x_ was deposited onto an Mo bottom electrode using Reactive Magnetron Sputtering. For both samples we see a high concentration of hydrogen in the SiO_x_: 16.3 at.% for sample 1 (ALD) and 5.9% for sample 9 (sputtered). In both cases, this exceeds the 2 at.%. threshold established in section 1.3, suggesting that hydrogen will modify the electronic properties of the SiO_x_ and thus affect ReRAM operation. Notably, there is a much higher concentration of hydrogen in the SiO_x_ deposited by ALD compared to that using sputtering. This is likely to arise from the hydrogen‐containing tris(dimethylamino)silane precursor used in ALD. However, despite this higher level of hydrogen, the sample 1(ALD) devices are harder to electroform with only 1 out of 32 measured devices successfully electroforming compared to 30 out of 32 for the sample 9 (sputtered) devices. IV sweeps for two devices per sample are shown in **Figure** **3**a. The ALD devices do not electroform even at −15 V, while both the sample 9 (sputtering) devices readily electroform below ‐2V. Results from two devices from sample 11 are also shown in Figure 3a for comparison which have identical top and bottom electrodes to the sample 1 (ALD) and the same thickness of SiO_x_ but instead deposited with sputtering. These readily electroform, confirming that the poor electroforming performance for sample 1 is a function of the ALD used for deposition. Previous work has shown that sputtered SiO_x_ grows with a columnar microstructure templated from the Mo bottom electrode which provides defect‐rich channels that enhance the switching properties of devices.^[^ ^7^ ^]^ In contrast, the ALD film is smoother, less defective, and requires ion implantation to induce a high enough defect density for electroforming.^[^ ^51^ ^]^ Thus, though hydrogen can generate defects in oxide thin films, for the ALD SiO_x_, in the absence of structural defects, the much higher concentration of hydrogen measured here is not sufficient to enable the devices to readily electroform and switch. The hydrogen may instead be passivating defects, as has previously been observed in the gate oxide community, which could explain why the sample 1‐ALD devices would in general not electroform.^[^ ^11^, ^12^ ^]^

Figure 2 shows that hydrogen is present not only in the SiO_x_ but also the electrodes. There is a significantly lower concentration of hydrogen in the Mo for sample 1 (1.3 at.%) than in sample 9 (5.9 at.%). This may arise from the high processing temperature used to deposit the SiO_x_ with ALD: 200 °C for 8 h, which effectively anneals the electrode, removing hydrogen. A surprising result was that for both sample 1 (ALD) and sample 9 (sputtered) there is a high level of oxygen in the Mo electrode at levels >10 at.%. This will impact the Mo performance as an oxygen reservoir, inhibiting it from storing and releasing oxygen during operation.^[^ ^52^ ^]^ Preventing this oxidation may further improve device performance.

### 2.3 Oxidation and Impurities

To investigate the oxidation of the electrodes revealed above, IBA and XPS measurements are compared for three samples which have electrodes with varying reactivity to oxygen. Samples 3, 4, and 5 have a 12 nm thick layer of sputtered SiO_x_ deposited onto Pt, Mo, and Ti electrodes, respectively. The IBA analysis, shown in Figures S1–S3 (Supporting Information), reveals an increase of electrode oxygen concentration increasing with electrode oxygen affinity: 2.4 at.% in the Pt electrode, 15.9 at.% in the Mo electrode, and 51.0 at.% in the Ti electrode.

Figure 3a shows a combined XPS depth profile of composition for all three samples, which supports the findings of the IBA, showing negligible levels of oxidation in the fairly inert Pt electrode but significant levels of oxidation throughout all the measured depths into the more reactive Mo and Ti electrodes. This confirms that when metals that readily oxidize are used as electrode materials which is often desirable for the active electrode in bipolar ReRAM devices — they may already be significantly oxidized in the pristine state post‐fabrication unless preventative measures are taken. The XPS depth profiles shown in Figure 3 also reveal a few nanometres of surface contamination, which supports the results of the IBA analysis in Figures S1–S3 (Supporting Information).

The findings of the IBA analysis shown in Figure 2 and the XPS shown in Figure 3 high levels of hydrogen throughout the samples, unintentional oxidation of metal electrodes, and significant impurity concentrations of carbon and nitrogen were consistent across many measured devices (Figures S1 and S12, Supporting Information), including those fabricated at a research foundry. Clearly, this will impact their performance, but how are the impurities introduced into devices? A low level of contamination is difficult to avoid in vacuum systems such as those used to deposit the samples in this work.^[^ ^54^ ^]^ Hydrogen, in particular, is notoriously mobile and difficult to remove from vacuum systems and is highly soluble in many of the metal targets used to deposit the electrodes, as well as the exposed surfaces of the equipment and vacuum chambers used during fabrication.^[^ ^31^ ^]^ The oxidation of electrodes could be a product of exposure to ambient between processing steps, oxygen plasma exposure during the SiO_x_ deposition, or gettering of oxygen from the SiO_x_ post‐fabrication. Notably, where oxygen is measured by IBA throughout Mo and Pt electrodes (Figures S1,  S2,  S4, and S6, Supporting Information), the oxidation is often only present in the top few nanometres of Ti electrodes (Figures S3,  S7, and S10, Supporting Information). Ti is known to form a tightly bound surface oxide, which likely prevents any subsequent oxidation.^[^ ^53^ ^]^ As these thin film samples are exposed to ambient conditions between fabrication and analysis, some impurities will also come from surface contamination. Across all measured samples the only layer free from contamination was the silicon wafer; this provided a good control to measure the effect of surface contamination. IBA analysis of a Si wafer surface, exposed to the same ambient conditions as the other samples, revealed a 9 nm thick native oxide containing 18.18 at% H and 5.45 at% C. Beneath this, the bulk Si had no measurable contaminants. This confirms that any thin film, no matter how pure, will experience a degree of surface contamination when exposed to an ambient environment. However, the impurities in devices measured here are not only present at the surface, but throughout the active layers of the devices, in high concentrations at the reactive interfaces where resistance switching is mediated.

The 15 devices for which IBA analysis was carried out here (Figure 2; Figures S1–S12, Supporting Information) show clear correlations between fabrication method and impurity concentration. The high temperatures used to deposit SiO_x_ with ALD appear to anneal hydrogen from the bottom electrode. The hydrogen‐containing precursors used during ALD or HSQ depositions lead to higher concentrations of hydrogen in the deposited SiO_x_ than in layers fabricated by sputtering from a silicon target in an oxygen atmosphere. Samples 11–14 (Figures S8–S11, Supporting Information), which were deposited by reactive sputtering, show high hydrogen concentrations in their TiN electrodes. Depositing thicker TiN electrodes onto Ru leads to a higher measured concentration of hydrogen in the Ru, suggesting some diffusion of hydrogen from the TiN during the deposition. Across the samples measured the more inert electrode materials, such as Au and Pt, remain metallic where the more reactive materials such as Ti and Mo oxidise readily during deposition.

Even if hydrogen is required for ReRAM operation, it will be necessary to prevent it being exchanged with the ambient environment during device operation as this would lead to performance varying with ambient humidity, which has previously been observed and remains problematic.^[^ ^22^, ^26^ ^]^ For gate oxides, the use of oxynitride barriers has been suggested to minimize hydrogen motion, prevent trap creation, and extend device lifetime.^[^ ^55^ ^]^ In our IBA measurements (Figures S3,  S5, and S7, Supporting Information) the only deposited thin films not containing measurable levels of hydrogen were those beneath a layer of TiO_2_. TiO_2_ could therefore act as a barrier layer: it is known to form a tightly bound oxide and has a hydrogen diffusion rate three orders of magnitude lower than that of metallic Ti.^[^ ^53^ ^]^ Metal oxides, in particular Al_2_O_3_, are used as hydrogen diffusion barriers in the nuclear fusion industry due to their low hydrogen permeability.^[^ ^56^ ^]^ As various metal oxides are already used in ReRAM devices, it should be relatively simple to implement a hydrogen capping layer.

Overall, to reduce the level of electrode oxidation, less reactive metals can be used, while samples should be kept in high vacuum throughout fabrication, and cross‐interface chemistry needs to be considered to prevent oxygen gettering. Similar considerations – avoiding breaks in the vacuum as well as avoiding certain solvents can help reduce other impurities, such as carbon. To reduce the concentration of hydrogen in samples it is important to use metallic targets with a low hydrogen solubility, avoid the use of hydrogen‐containing solvents, add annealing steps, and seal devices with hydrogen diffusion barriers. It is likely that these two final steps will be the most crucial, as it may be very challenging to entirely prevent hydrogen from entering the fabrication process at all stages. Annealing can be used to lower the level of hydrogen to a desirable concentration and then the diffusion barrier will prevent any further reactivity with the external environment post‐fabrication. By systematically measuring and controlling each stage at which impurities are introduced into the system it will be possible to further improve the control of oxygen, hydrogen, and other contaminants in pristine devices, likely greatly benefiting the consistency and performance of ReRAM devices.

### 2.4 Hydrogen Diffusion

**Figure 4:**
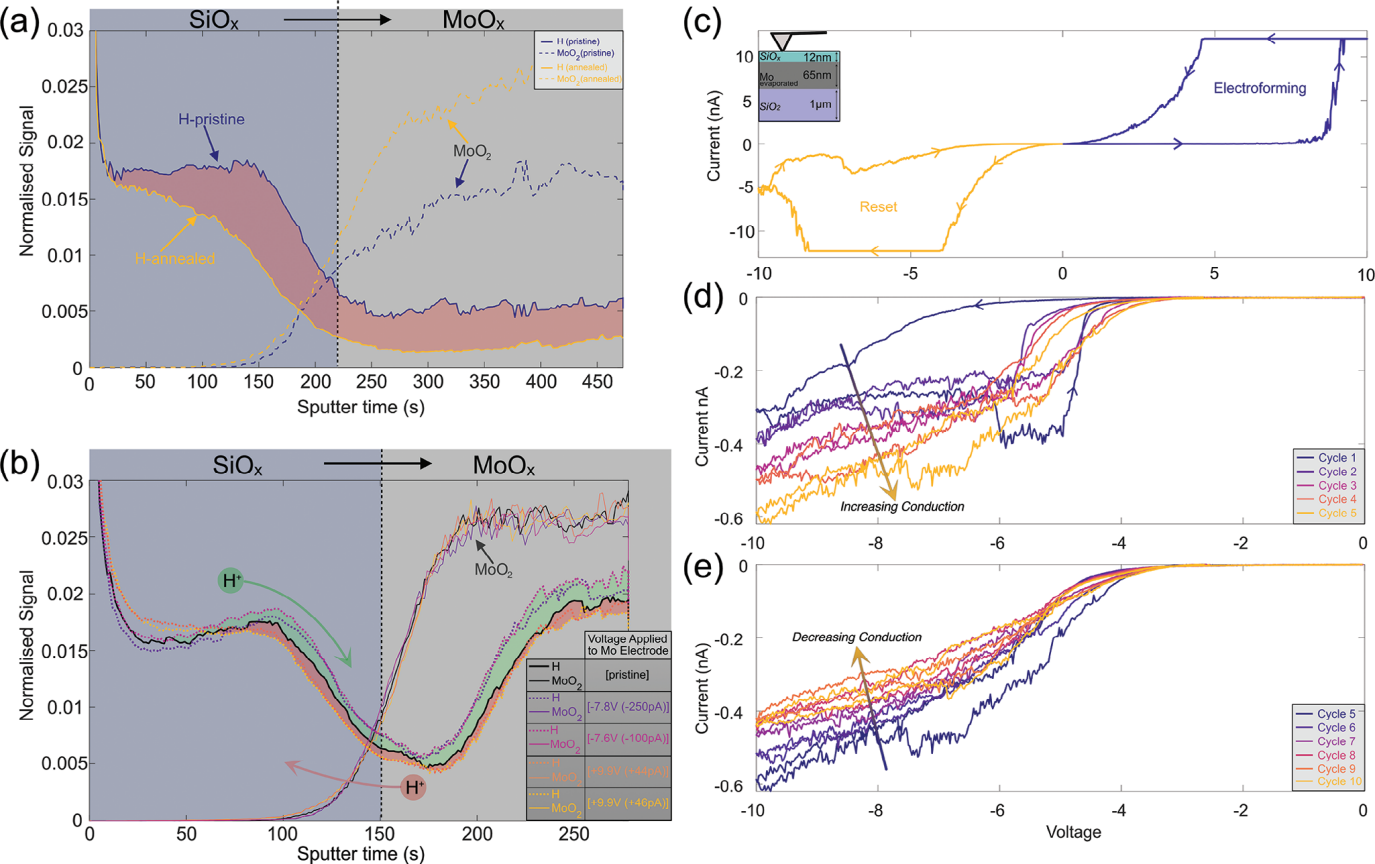
a) SIMS depth profiles normalized to the total ion signal for pristine HSQ SiO_x_ samples before and after annealing revealing a loss of hydrogen during annealing. IBA analysis of the annealed sample (sample 6) is shown in Figure S4 (Supporting Information). b) SIMS depth profiles normalized to the total ion signal comparing the hydrogen concentration in sample 4 between pristine devices as well as devices electrically biased with opposite polarities. Increases and decreases in the hydrogen concentration at each depth are illustrated with green and red shading, respectively. This illustrates the reversible field‐driven diffusion of hydrogen to and from the interface under negative and positive voltages applied to the bottom electrode. Corresponding IBA analysis is found in Figure S2 (Supporting Information). Forward and reverse IV sweeps carried out on a comparable SiOx sample to that in b) show: c) electroforming and resetting, d) an initial increase in conduction from cycles 1–5 with a negative bottom electrode bias, e) a decrease in conduction under the same bias from cycles 5–10.

Having confirmed the presence of high concentrations of hydrogen in pristine devices it is important to understand its role in device operation. During the reset process, oxide films in ReRAM devices are subjected to localized elevated temperatures due to Joule heating, and high applied fields. Both will affect the hydrogen distribution. To measure this, SIMS depth profiles of several devices with molybdenum bottom electrodes are compared below in **Figure** **4**. The measured signals are normalized to total ion count to reduce matrix effects and account for variations between the measurements.^[^ ^57^ ^]^ The MoO_2_ signals illustrate the position of the interface between SiO_x_ and the Mo bottom electrode.

In Figure 4a the depth profiles show the effect of annealing at 160 °C for 2 min on the hydrogen distribution within a pristine, unbiased silicon oxide film fabricated from spin‐on hydrogen silsesquioxane (HSQ).^[^ ^28^ ^]^ Annealing reduces hydrogen concentration throughout the oxide and electrode, confirming hydrogen redistribution at temperatures lower than that reached during ReRAM operation and matching findings from the gate oxide community.^[^ ^38^ ^]^

To measure the effect of an applied field on the hydrogen distribution in devices a new experimental procedure, detailed further elsewhere,^[^ ^52^ ^]^ was used combining Conductive Atomic Force Microscopy (CAFM) and SIMS to provide a robust comparison of ion concentrations before and after electrically biasing a sample. In Figure 4b depth profiles are carried out through several different devices in sample 4, showing the measured hydrogen signal at each depth, which is normalized to the total measured ion signal to enable a robust comparison. The green (red) highlighted regions illustrate an increase (decrease) in the measured hydrogen concentration for electrically biased devices when compared to a reference pristine device. This reveals that applying a negative (positive) bias to the Mo bottom electrode causes an increase (reduction) in the hydrogen concentration at the SiO_x_/Mo interface. This confirms, for the first time, the reversible diffusion of hydrogen to and from the interface during ReRAM operation. Using the same CAFM set up, IV sweeps were carried out on a comparable sputtered SiO_x_ sample and are shown in Figure 4c–e.^[^ ^58^ ^]^ Figure 4c shows conventional operation where the device is electroformed with a positive Mo bias and reset with a negative Mo bias. Comparing this to Figure 4b, during electroforming (where the conductivity of the device is increased as a conductive oxygen vacancy filament is formed) there is a reduction in hydrogen concentration at the SiO_x_/Mo interface; during the reset (where the conductivity of the device is decreased as the oxygen vacancy filament is locally disrupted) there is an increase in hydrogen concentration at the interface. This could indicate that hydrogen passivates defects at the interface. We cannot, however, decouple the influence of hydrogen from the simultaneous diffusion of oxygen, which has previously been measured and reliably explains changes in conductivity.^[^ ^52^ ^]^ However, Figure 4d,e shows a deviation from this typical behavior. On applying a series of negative bias sweeps to the Mo electrode the conductivity at first increases and then, after the fifth cycle, begins to decrease again. This behavior cannot be explained by oxygen diffusion alone, nor from Joule heating as the measured currents are an order of magnitude lower than those observed during the electroforming, in Figure 4c. This suggests that the oxide is not breaking down completely through the formation of a conductive filament. Instead, it suggests the presence of competing mechanisms, perhaps those of hydrogen and oxygen diffusion. The initial increase in conductivity during cycles 1–5 could be from the removal of oxygen from the SiO_x_/Mo interface, previously measured for this sample.^[^ ^52^ ^]^ After this, during cycles 5–10, the hydrogen diffusion to the SiO_x_/Mo interface shown in Figure 4b may passivate the oxygen vacancies and subsequently reduce the oxide conductivity.

Overall, our results reveal that both elevated temperatures and high applied electric fields can induce measurable hydrogen redistribution during ReRAM operation. Thus, the dual role of hydrogen and oxygen diffusion must be considered in models of resistance switching and the design of ReRAM memristive systems.

## 3 Conclusion

Hydrogen has been shown previously to influence strongly the conductivity of transistor gate oxides – the same oxides used in ReRAM – above a critical threshold concentration of 2 at.%. Quantitative IBA measurements on our ReRAM devices reveal hydrogen concentrations considerably exceeding this threshold in all samples measured. This suggests that hydrogen likely plays a role in resistive switching. IV measurements show that, in the absence of structural defects, in an ALD‐deposited SiOx thin film, the presence of hydrogen is not sufficient to induce switching. IBA analysis also showed significant oxidation of electrodes and some carbon and nitrogen contamination which, if controlled, could improve the effectiveness of the electrodes as oxygen reservoirs. The contamination correlates with the fabrication technique. Our results also suggest that TiO_2_ is a barrier to hydrogen diffusion which, along with other suitable oxides such as Al_2_O_3_, could contain hydrogen during operation and prevent exchange with the environment.

Our SIMS measurements reveal that hydrogen is mobile under ReRAM operating conditions: diffusing out of samples at elevated temperatures and diffusing to and from the SiOx/Mo interface under applied electric fields. Our results suggest that hydrogen could be passivating defects in our devices. However, this likely depends on concentration and operating conditions as hydrogen can both increase or decrease oxide conductivity.

In summary, hydrogen is present, impacts the electronic properties of and diffuses through ReRAM devices during operation. The results shown here provide confirmation that hydrogen needs to be incorporated into models of ReRAM operation and much more tightly controlled during fabrication and operation. It may be detrimental or essential for ReRAM operation; either way it cannot be ignored. Further detailed experimental studies are needed to provide the basis for much‐needed models for the role of hydrogen in resistance switching in oxides.

## 4 Experimental Section

### Fabrication

Several types of structures and devices are discussed in this work:

Samples 1 to 11:

These structures were deposited on SiO_2_/Si substrates. The bottom electrodes (Mo, Ti, Pt) were deposited either by magnetron sputtering or thermal evaporation. The SiOx switching layers were deposited by one of the following methods: 1. Reactive magnetron sputtering in an argon and oxygen ambient. 2. Atomic layer deposition (ALD) with tris(dimethylamino)silicon (TDMAS) and ozone as precursors. The deposition temperature was 200 °C. 3. Spin‐coating with an HSQ solution. After spin‐coating, the HSQ film was annealed on a hot plate at 160 °C to remove the solvent.

To complete the devices, a Ti/Au top electrode was deposited onto the SiO_x_ layers by thermal evaporation. A shadow mask was used to define the device sizes, which range from 200um x 200um to 800um x 800um.

Samples 12 to 15:

These TiN structures were deposited on SiO_2_/Si substrates by magnetron sputtering. Before the TiN was grown, a TaN/Ru layer was deposited onto the SiO_2_/Si substrate.

### Secondary Ion Mass Spectrometry

Depth profiling was carried out using IONToF ToF‐SIMS V instrument with a 1 keV Cs^+^ sputter beam and a 25 keV Bi^+^ analysis beam, both at 45° to the sample surface. The instrument is operated in a Burst Alignment setting in the burst mode, in which the primary ion beam pulse is chopped up into 7 shorter bursts. This provides a good compromise between lateral resolution (300 nm) and mass resolving power (≈10 000). Surface or interface non‐uniformity and ion mixing from the Cs sputtering beam, which is determined by its ion penetration depth, will result in some interface broadening and hence slightly reduced depth resolution.^[^ ^59^ ^]^ As the Cs beam is low energy and at a 45° angle, R_p_ ≈ 4.4 nm. However, there will also be cascade mixing and recoil implantation from the Bi primary ion beam, which has a much higher energy. Cascade mixing of layers is the more dominant of the two effects and will occur over a distance on the order of the primary ion range, R_p_.^[^ ^60^ ^]^ Using SRIM Monte Carlo modelling software this range was calculated for the samples as R_p_ ≈ 13 .4nm. This could result in interface broadening. However, the low beam fluence means that very few ions will reach the sample interface. The Cs sputtering beam will therefore produce the more dominant mixing effect.

### X‐ray Photoelectron Spectroscopy

A Thermo Fisher K‐Alpha+ high‐throughput x‐ray photoelectron spectrometer was used to collect survey spectra whilst depth profiling through the sample. This instrument features a monochromated, microfocused Al Kα X‐ray source, hν = 1486.7 eV, and a 180° double‐focusing hemispherical analyzer equipped with a 2D detector. The X‐ray source operated at an emission current of 6 mA and an anode bias of 12 kV, resulting in an X‐ray spot size of 400 µm^2^. Survey spectra were acquired using 200 eV pass energy. Depth profiling was carried out using a 500 eV Ar^+^ sputter gun at 30° with a 2 mm^2^ crater size and 30s per etch cycle. The Ar^+^ sputtering used is relatively inert, which minimizes beam‐induced material segregation material. A survey spectrum was taken at each depth, with limited energy resolution, therefore the composition can be measured but not the material's oxidation state. This was chosen to reduce the time required for each measurement and because of concern that Ar sputtering might reduce the surface of the SiO_x_ and skew any measurements of oxidation. A flood gun was used to compensate for sample charging. Thermo Advantage software was used to process and collect the data; a Shirley background was subtracted from the survey spectra before calculating the relative atomic percentages of each element; MATLAB R2020b was used to plot the data.

### Time of Flight Elastic Recoil Detection Analysis & Rutherford Backscattering Spectrometry

For the ToF‐ERDA measurements, the beam line was used at the University of Jyväskylä. The cesium sputter ion source (SNICS) was used to generate negative Cl and I ions, formed due to cesium ion bombardment. A 1.7 MV Pelletron accelerator generated the ion beam at the correct energy: 10 or 10.2 MeV ^35^Cl^5+^, 15.3 or 16 MeV ^127^I^8+^. After sputtering, the ions are negatively charged; the accelerator applies a voltage to accelerate them to the correct energy and switch them to positively charged.^[^ ^61^ ^]^ The Potku software used to analyze the measurements contains a program that simulates the ToF‐ERDA process, called Monte Carlo elastic recoil detection (MCERD). The simulated spectrum was overlapped with the experimentally measured spectra and used to account for effects including beam spot size, multiple and plural scattering, and scattering in the detector foils.^[^ ^47^ ^]^ However, there was greater uncertainty in the results of ToF‐ERDA for heavier elements, especially when the element in question is heavier than the incident beam, thus RBS measurements were carried out to complement the ToF‐ERDA results.^[^ ^42^ ^]^ For the RBS measurements, the beam line at the Surrey Ion Beam Centre was used. A HV 358 Duoplasmatron ion source was used, with a He gas bottle as an ion source. Helium ions were negatively charged. The 2 MV Tandem accelerator was then used to select the correct energy; with a 2.5 mm aperture selected for the beam spot size down the beam line. The ions then entered the sample chamber, where there is a 6‐axis goniometer for sample movement and 2 Passivated Implanted Planar Silicon (PIPS) RBS detectors (1 in IBM geometry, (det B) and 1 in Cornell geometry, (det A); see fig 3.17 in SIMNRA user guide for details.^[^ ^62^ ^]^

### Conductive Atomic Force Microscopy

CAFM measurements were carried out under ambient conditions using a Bruker Icon AFM and Pt/Ir‐coated silicon probes (Spark, NuNano). In Figure 4c) the CAFM is operated in the 12.5nA range thus the detector is saturating at higher voltages. Therefore, though the displayed current is 12.5nA in certain ranges, the true current through the device will be higher.

## Conflict of Interest

Two of the authors, Anthony J. Kenyon and Adnan Mehonic, are co‐founders of Intrinsic Semiconductor Technologies ltd.

## Supporting information



Supporting Information

## Data Availability

The data that support the findings of this study are available from the corresponding author upon reasonable request.
