# Supplementary material for: The Role of Hydrogen in ReRAM
Source: Adv Mater. 2024 Oct 14;36(52):2408437. doi: 10.1002/adma.202408437 (PMC11681311; doi:10.1002/adma.202408437)
Supplement: Supplementary file 1 — Supporting Information [file ADMA-36-2408437-s001.docx]

Supporting Information

Title ((SI - The Role of Hydrogen in ReRAM ))

Horatio R. J. Cox*, Matthew K. Sharpe, Mikko Laitinen, Callum McAleese, Jeevan Dulai, Richard Smith, Jonathan England, Wing H. Ng, Mark Buckwell, Longfei Zhao, Sarah Fearn, Adnan Mehonic, Anthony J. Kenyon

All the measured devices shown here in Figures S1-S12 have hydrogen contamination throughout most of the layers and some carbon/nitrogen contamination. This contamination appears to be a problem at the interfaces, likely due to breaks in the vacuum and ambient exposure between layer depositions.

In Figures S1-S3 high levels of hydrogen are measured throughout the evaporated Pt electrode, the sputtered Mo electrode, and the sputtered Ti electrode. Notably, one of the Ti layers measured in S3 has a measured hydrogen concentration of zero. Indeed, the only layers without hydrogen — other than the SiO_2_/Si substrate — are in devices containing Ti (S3/S5/S7). We speculate that this could be a product of a tightly bound TiO_2_ layer, formed at the surface of the Ti, which acts as a hydrogen permeation barrier. However, the limited depth resolution of the IBA analysis, combined with surface roughness, means that results from each layer will have some contribution from the adjacent layer. This makes it hard to be certain of the Ti stoichiometry at the interfaces other than to say it is generally oxidised.


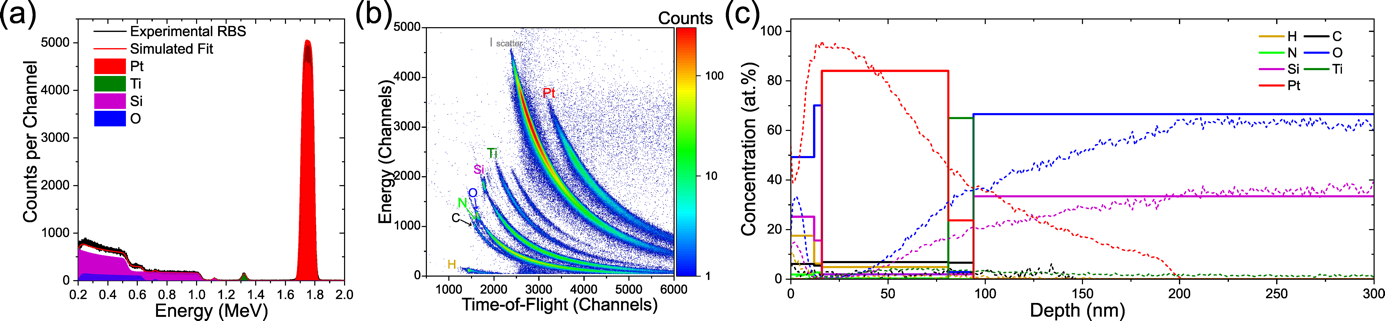


**Figure S1 combined IBA analysis of Sample 3**. a) RBS spectrum b) ToF-ERDA histogram. c) Dotted line showing ToF-ERDA depth profile; solid line showing the final composition obtained from the combined IBA analysis.


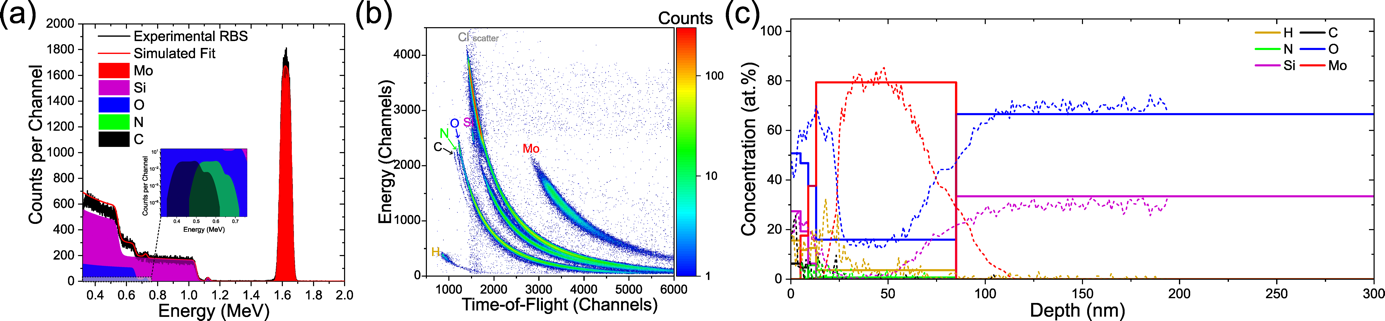


**Figure S2 combined IBA analysis of Sample 4**. a) RBS spectrum b) ToF-ERDA histogram. c) Dotted line showing ToF-ERDA depth profile; solid line showing the final composition obtained from the combined IBA analysis.


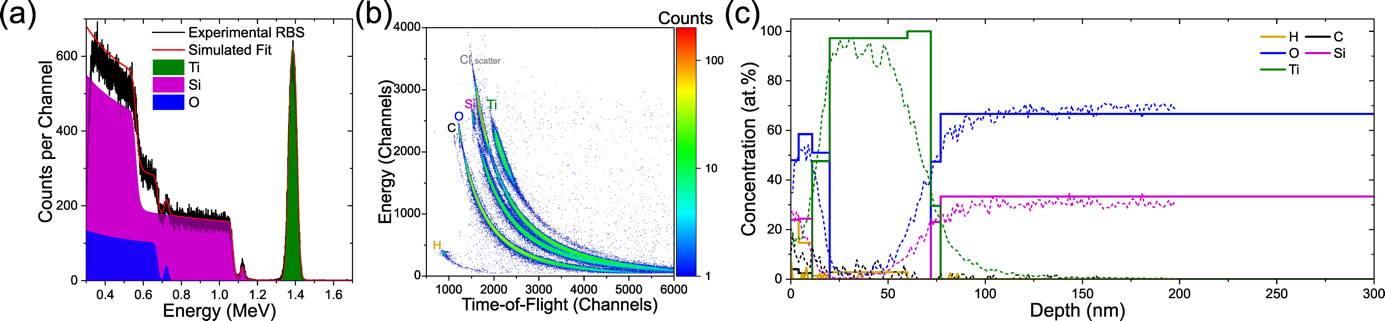


**Figure S3 combined IBA analysis of Sample 5**. a) RBS spectrum b) ToF-ERDA histogram. c) Dotted line showing ToF-ERDA depth profile; solid line showing the final composition obtained from the combined IBA analysis.

In Figure S4, hydrogen silsesquioxane (HSQ) was spin coated and annealed at 160 ºC for two minutes to form a networked structure of amorphous silicon oxide. The HSQ has a general formula HSiO_3/2_)_2n_, according to which there should be approximately 29 at.% hydrogen. We measure a concentration of 18.6 at.% confirming that some hydrogen is removed from the sample during annealing. This is confirmed by SIMS measurements taken before and after annealing, shown in Figure 4 a). The HSQ is delivered in a carbon-containing methyl isobutyl ketone (MIBK) solvent, which explains the levels of carbon measured in the SiO_x_.


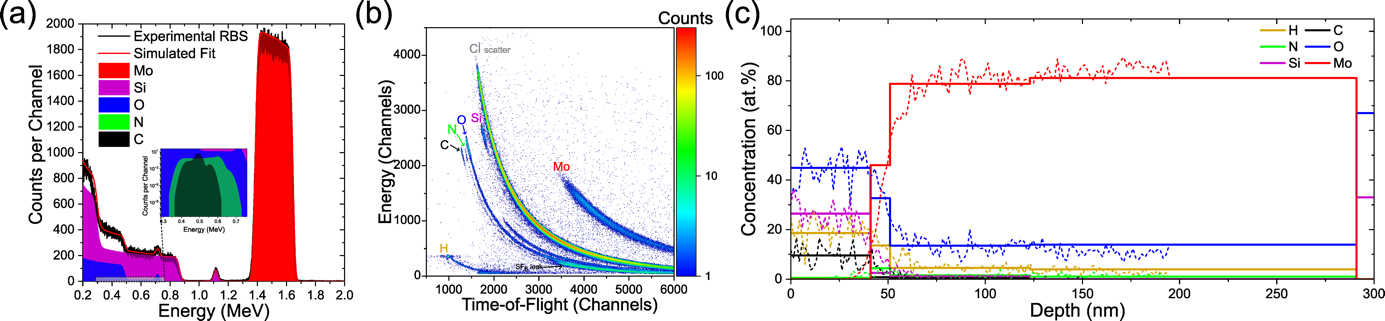


**Figure S4 combined IBA analysis of Sample 6**. a) RBS spectrum b) ToF-ERDA histogram. c) Dotted line showing ToF-ERDA depth profile; solid line showing the final composition obtained from the combined IBA analysis.


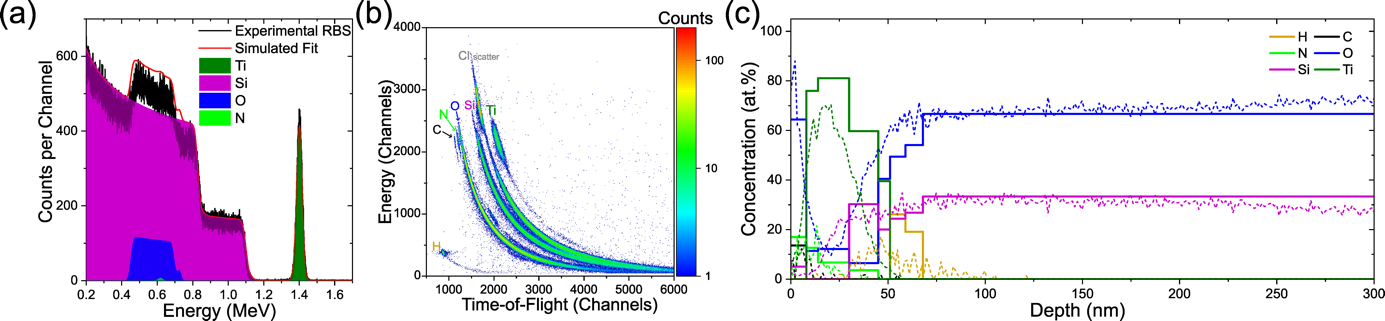


**Figure S5 combined IBA analysis of Sample 8**. a) RBS spectrum b) ToF-ERDA histogram. c) Dotted line showing ToF-ERDA depth profile; solid line showing the final composition obtained from the combined IBA analysis.


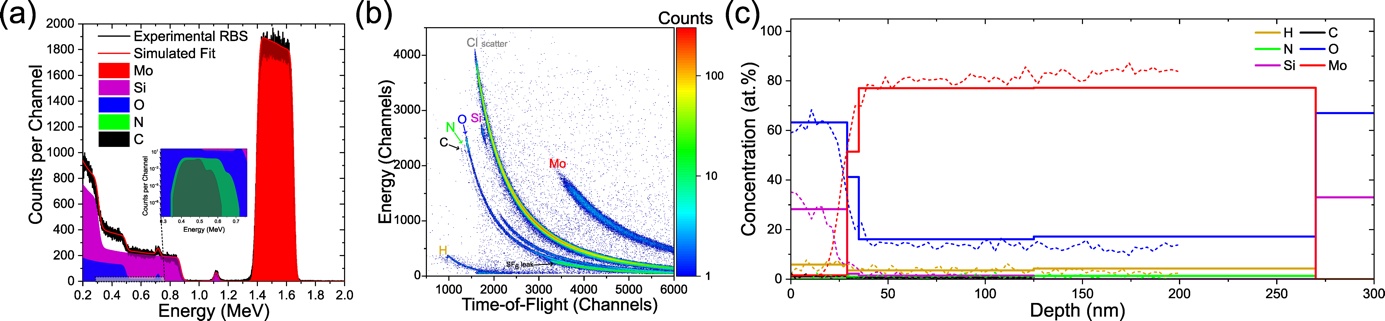


**Figure S6 combined IBA analysis of Sample 9**. a) RBS spectrum b) ToF-ERDA histogram. c) Dotted line showing ToF-ERDA depth profile; solid line showing the final composition obtained from the combined IBA analysis.


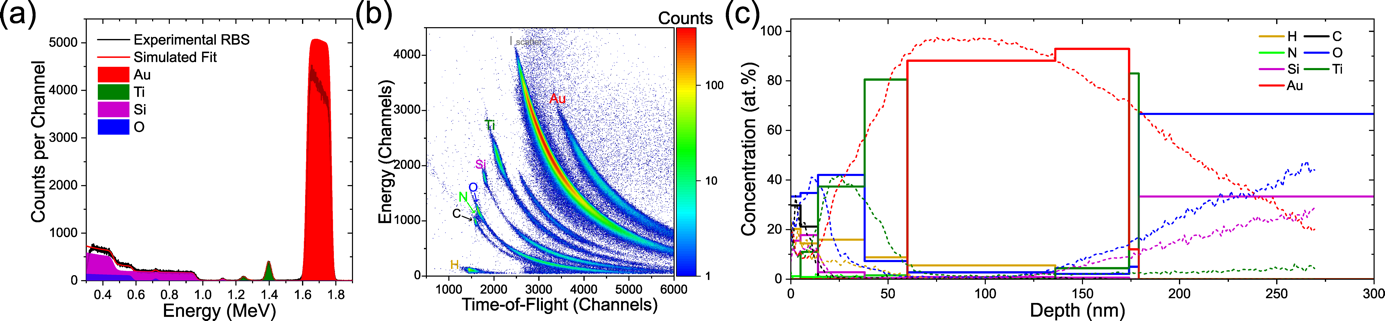


**Figure S7 combined IBA analysis of Sample 10**. a) RBS spectrum b) ToF-ERDA histogram. c) Dotted line showing ToF-ERDA depth profile; solid line showing the final composition obtained from the combined IBA analysis.

The devices measured thus far were all fabricated academic research clean room facilities, at

the London Centre for Nanotechnology, where many different types of sample are processed. As the Si and SiO_2_ layers measured on the commercially fabricated wafers are measured to be extremely pure, it was expected that the same would apply to devices fabricated in a foundry. However, Figures S8-S11 are measurements of ReRAM thin film layers deposited at a research foundry under much more stringently controlled conditions. The measured concentration of hydrogen is very high throughout the device layers. Clearly, without explicitly controlling for hydrogen, it is likely to be present at a significant contaminant for ReRAM technologies.


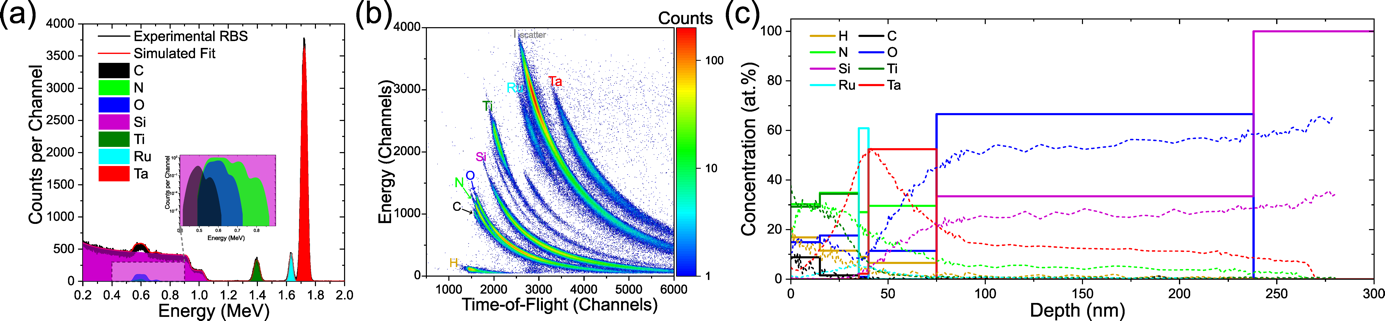


**Figure S8 combined IBA analysis of Sample 12**. a) RBS spectrum b) ToF-ERDA histogram. c) Dotted line showing ToF-ERDA depth profile; solid line showing the final composition obtained from the combined IBA analysis.


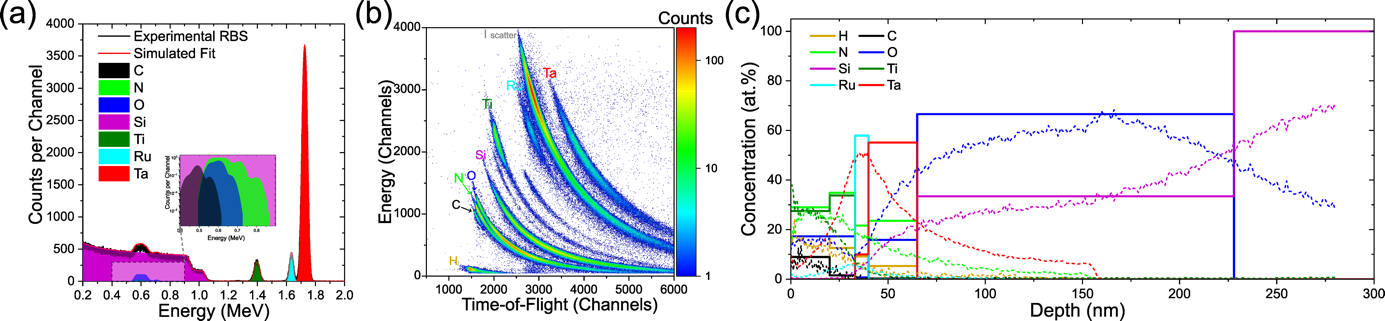


**Figure S9 combined IBA analysis of Sample 13**. a) RBS spectrum b) ToF-ERDA histogram. c) Dotted line showing ToF-ERDA depth profile; solid line showing the final composition obtained from the combined IBA analysis.


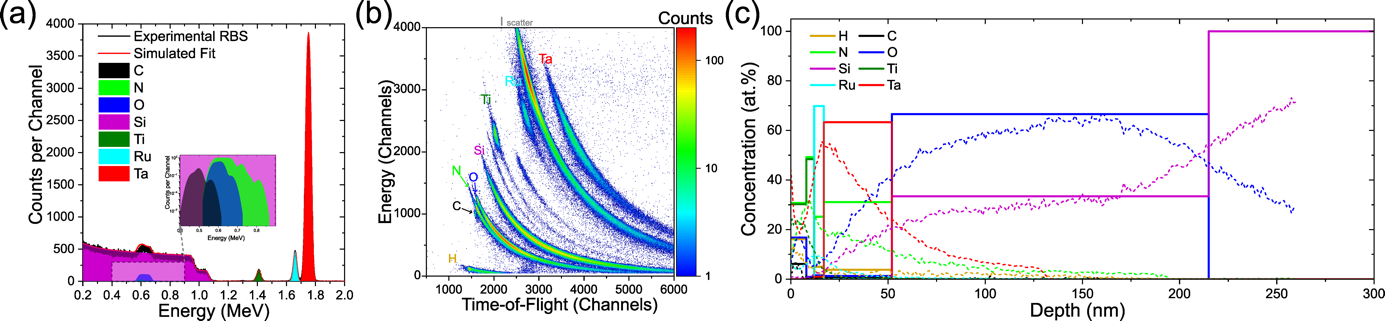


**Figure S10 combined IBA analysis of Sample 14**. a) RBS spectrum b) ToF-ERDA histogram. c) Dotted line showing ToF-ERDA depth profile; solid line showing the final composition obtained from the combined IBA analysis.


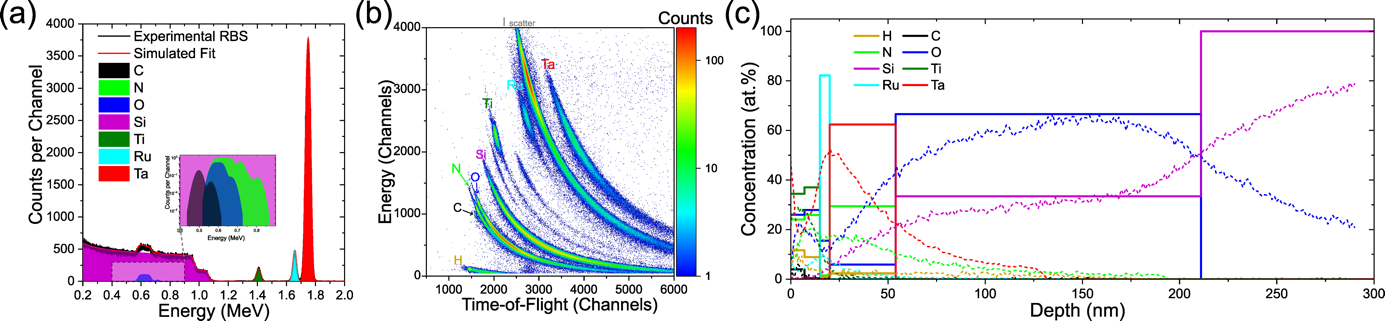


**Figure S11 combined IBA analysis of Sample 15**. a) RBS spectrum b) ToF-ERDA histogram. c) Dotted line showing ToF-ERDA depth profile; solid line showing the final composition obtained from the combined IBA analysis.


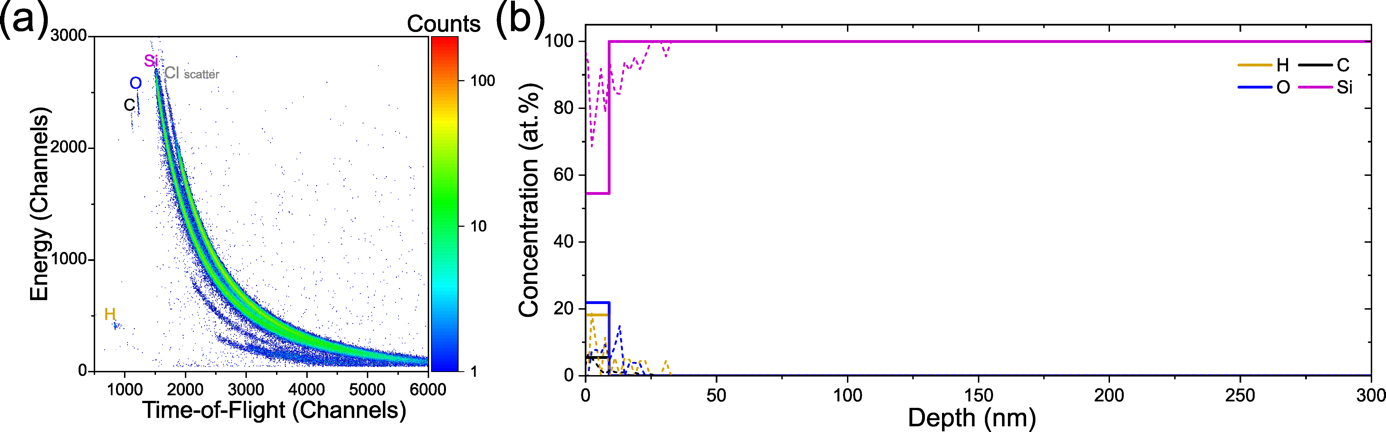


**Figure S12 combined IBA analysis of Sample 16**. a) ToF-ERDA histogram. b) Dotted line showing ToF-ERDA depth profile; solid line showing the final composition obtained from the MCERD corrected fit.

Samples 3-5, 8, 10, 12-15 were analysed with ToF-ERDA at the Surrey Ion Beam Centre. The measurement principle is illustrated in Figure S13. We used a HV 860C multi sample sputter source, using solid targets to create ions: AgCl and either CsI or AgI for Cl and I, respectively. A 2MV Tandem accelerator generated the ion beam at the correct energy: 10 or 10.2 MeV ^35^Cl^5+^, 15.3 or 16 MeV ^127^I^8+^. Samples 1, 2, 6, 9 were measured at Jyvaskyla using the ToF-ERDA set up illustrated in Figure 1 and detailed in the experimental methods of the main paper.


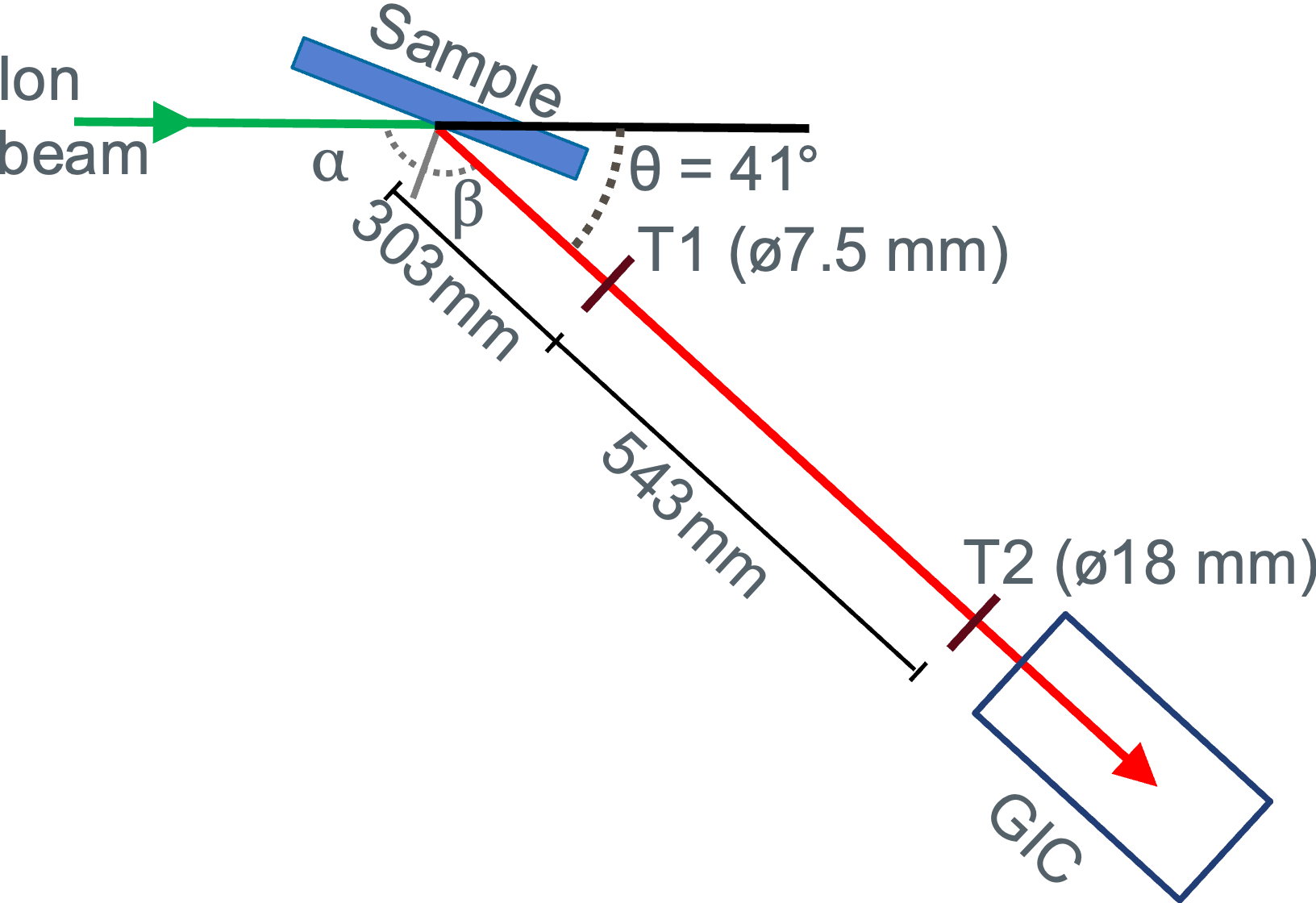


**Figure S13 ToF-ERDA schematic.** Schematic of the ToF-ERDA set up used at the Surrey Ion Beam Centre with α/β = 69.5º. This was used to analyse samples 3-5, 8, 10,12-16. Samples 1, 2, 6, 9 were measured at Jyvaskyla using the ToF-ERDA set up illustrated in Figure 1.


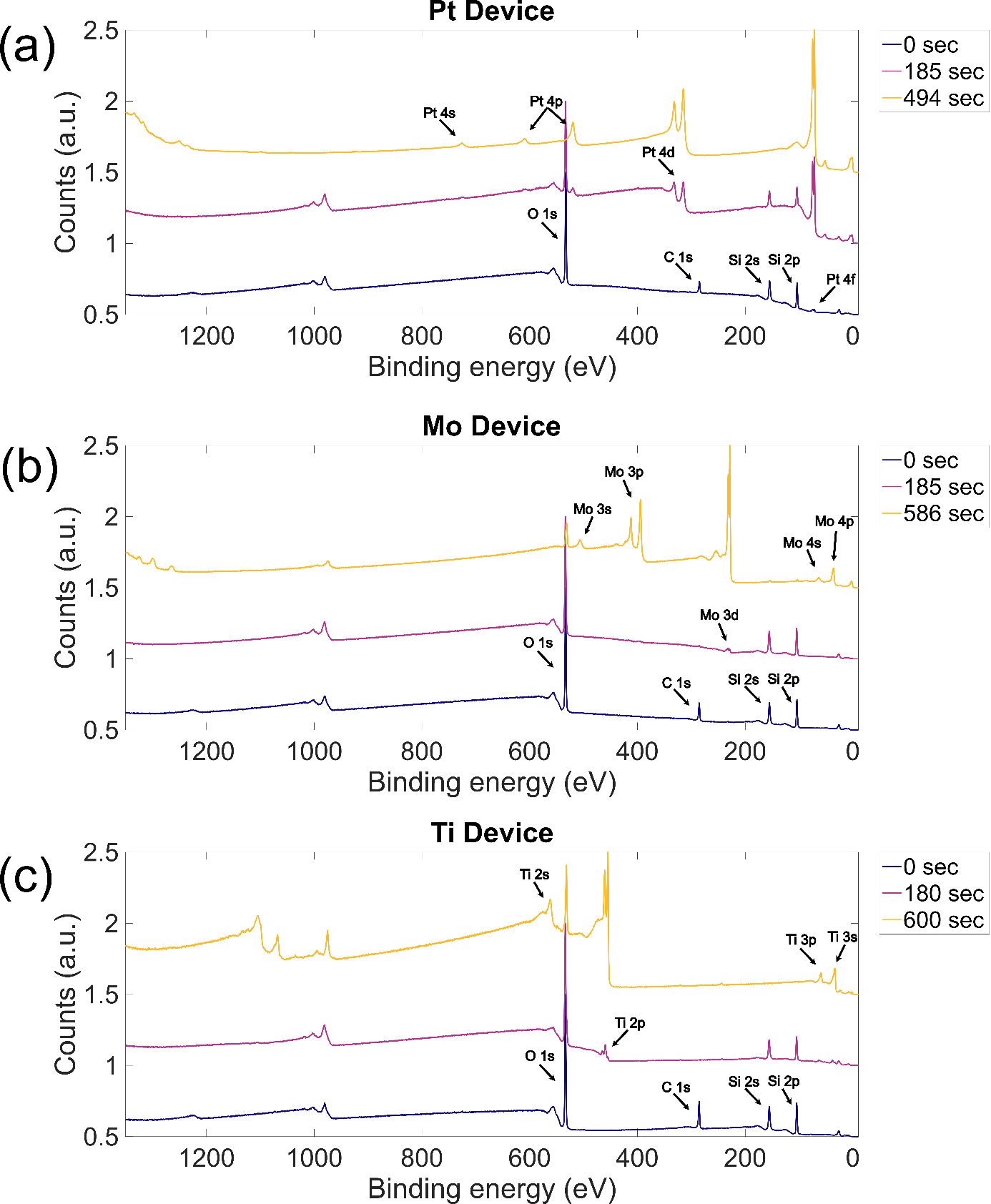


**Figure S14 XPS Survey Spectra.** a)-c) XPS survey spectra for the *Pt, Mo and Ti* devices (samples 3, 4 and 5) respectively. Spectra are shown for three different depths in the depth profile: one at the surface before sputtering commences, one at the SiO_x_ /metal interface and one in the metal electrode. Reproduced from [41] with permission of the author.
